# Ketamine impairs growth cone and synaptogenesis in human GABAergic projection neurons via GSK-3β and HDAC6 signaling

**DOI:** 10.1038/s41380-022-01864-5

**Published:** 2022-11-21

**Authors:** Xuan Li, Hexige Saiyin, Xinyu Chen, Qiong Yu, Lixiang Ma, Weimin Liang

**Affiliations:** 1 Department of Anesthesiology, Huashan Hospital, Fudan University, Shanghai, China; 2 Department of Anesthesiology, The Fifth Affiliated Hospital, Sun Yat-Sen University, Zhuhai, Guangdong China; 3 State Key Laboratory of Genetic Engineering, School of Life Sciences, Fudan University, Shanghai, China; 4 Department of Anatomy and Histology & Embryology, School of Basic Medical Sciences, Fudan University, Shanghai, China

**Keywords:** Neuroscience, Cell biology

## Abstract

The growth cone guides the axon or dendrite of striatal GABAergic projection neurons that protrude into the midbrain and cortex and form complex neuronal circuits and synaptic networks in a developing brain, aberrant projections and synaptic connections in the striatum related to multiple brain disorders. Previously, we showed that ketamine, an anesthetic, reduced dendritic growth, dendritic branches, and spine density in human striatal GABAergic neurons. However, whether ketamine affects the growth cone, the synaptic connection of growing striatal GABAergic neurons has not been tested. Using human GABAergic projection neurons derived from human inducible pluripotent stem cells (hiPSCs) and embryonic stem cells (ES) in vitro, we tested ketamine effects on the growth cones and synapses in developing GABAergic neurons by assessing the morphometry and the glycogen synthase kinase-3 (GSK-3) and histone deacetylase 6 (HDAC6) pathway. Ketamine exposure impairs growth cone formation, synaptogenesis, dendritic development, and maturation via ketamine-mediated activation of GSK-3 pathways and inhibiting HDAC6, an essential stabilizing protein for dendritic morphogenesis and synapse maturation. Our findings identified a novel ketamine neurotoxic pathway that depends on GSK-3β and HDAC6 signaling, suggesting that microtubule acetylation is a potential target for reducing ketamine’s toxic effect on GABAergic projection neuronal development.

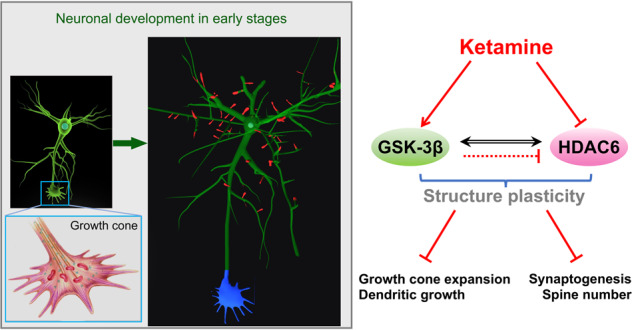

## Introduction

Axons or dendrites led by the growth cone navigate to their targeted sites, wire correct neuronal circuits, and proper synaptic contacts through regulated structural plasticity in the fetal brain and are involved in recovery after brain injury. Structural plasticity is evidenced by the structural changes of the growth cone and neurite, including length and count, and numerical changes of dendritic spines and synapse [1]. These cues in striatal projections neurons (SPNs) lead the correct projections to the globus pallidus (GPi) and the substantia nigra pars reticulata (SNr), wiring the most prominent descending longitudinal fiber systems in the forebrain [2]. The striatum balances the output through the intrinsic architecture of the striatal network, named the direct and indirect pathways [3, 4]. Aberrant integration of striatal projections into circuits can lead to a wide range of brain disorders with motor and cognitive symptoms, such as schizophrenia, autism spectrum disorder (ASD), Gilles de la Tourette syndrome, obsessive-compulsive disorder (OCD) and OC-spectrum disorders, and Huntington’s disease (HD) [5–12], highlighting the indispensable role of structural plasticity in SPNs. In a healthy individual, ketamine decreased hippocampal connectivity, including frontotemporal and temporoparietal functional connectivity [13]. In primate models, chronic ketamine exposure induces apoptosis and might create irreversible and long-term deficits in brain functions [14]. In rodent models, maternal ketamine exposure caused anxiety and depression-like behavior, and cognitive impairment in progeny [15], and crippled fetal brain development [16]; ketamine exposure in juveniles caused permanent and functional changes in the brain and disrupted the progression of normal developmental trajectories [17]. Our previous work showed that ketamine induces abnormal structural plasticity at anesthetic doses in hiPSC-derived striatal GABAergic projection neurons [18], which may be related to functional defects in the striatal circuits after anesthesia exposure [19–21]. To what extent ketamine impairs axon pathfinding and which cues are involved remain largely unknown. For example, the pathway through which ketamine affects the structural plasticity of SPNs is unknown.

Glycogen synthase kinase-3β (GSK-3β) regulates neurogenesis, axonal growth, microtubule dynamics, synaptic plasticity, and neuronal polarity [22–24]. Hyperphosphorylation of GSK-3β occurs in hippocampal neurons, and this phosphorylation-dependent inhibition of GSK-3β is essential for neuronal dendritic structure and neurite growth [25]. Inhibition of GSK-3β activity exerts a reliable neuroprotective effect at the onset of neuroinflammatory response-mediated neurodegenerative change [26]. In addition, overexpression of GSK-3β led to abnormal dendritic development and decreased the expression of synapse-associated proteins in hippocampal granule neurons of newborns [27]. These studies indicate that GSK-3β activity might play a pivotal role in neuronal development and maturation. Recent studies have shown that the abnormal structural development of GSK-3β knockout mice may be related to the kinetic changes resulting from phosphorylation of key microtubule regulatory proteins [26, 28]. In our previous study, HDAC6 was involved in ketamine-induced morphological impairments of SPNs by regulating their substrate α-tubulin [18]. It has been shown that GSK-3β can phosphorylate HDAC6 on serine 22 (Ser-22) sites, leading to the deacetylation of α-tubulin and breakdown of microtubule structure [29, 30]. Therefore, the present study investigated the effects of ketamine on structural plasticity in terms of GSK-3β mediated alteration of cytoskeletal dynamics.

Herein, we tested the role of GSK-3β and HDAC6 in ketamine-mediated structural plasticity in SPNs by evaluating the growth cone, total neurite length, primary neurite number, dendritic spine density, and the expression of synapse-associated proteins in human-induced GABAergic neurons. By using human inducible pluripotent stem cells (hiPSCs) derived from a healthy donor and embryonic stem cells (ES) and differentiated into SPNs, we not only showed that these models were suitable to test the effects and mechanism of clinical anesthetic agents in human neuronal development but also illustrated ketamine-impaired GABAergic neuronal axonal and dendrite guidance and synaptic networks by GSK3β- and HDAC6-mediated pathways.

## Materials and methods

See supplementary materials for additional details.

### Pharmacological agents

The pharmacological agents used in the present study are detailed in Supplementary Material I: Supplementary Table S1. The solvent required for the specific drugs was used for the active treatment at the same dilution for each vehicle treatment.

### Ethics and hiPSCs culture

Human iPSCs derived from 19-year-old females (8–12, derived from skin fibroblasts) and the H9 cell lines were cultured on Matrigel or Vitronectin-coated (Corning, NY, USA) plates in E8 medium (Gibco, Carlsbad, CA, USA). Floor-plate-based striatal SPNs inductions and dual-SMAD inhibition protocols were performed following the previous study [18]. The ethics of this HD work was approved (No. 28) by the Ethics Committee of the Institutes of Biomedical Sciences at Fudan University.

### Human fibroblasts and construction of hiPSC

The skin samples from the upper arm were collected by a skin punch (diameter, 0.3 × 0.3 cm) by a surgeon, and the skin was transferred to a Biosafety Cabinet, and cut into smaller fragments using surgical knives, plated on dishes using forceps, and cultured for several weeks to obtain enough fibroblasts. Fibroblasts were maintained in DMEM high glucose (Euroclone), 10% FBS (Euroclone), 2 mM L-glutamine (Euroclone, Italy), and 1% penicillin/streptomycin (Invitrogen). A total of 1.5 × 10^4^ fibroblasts were transfected with the Sandai virus construct that encoded the transcription factors OCT4, SOX2, and KLF4 (OSK, with or without C-MYC). The infected fibroblasts were replated on a feeder layer of mouse embryonic fibroblasts (MEFs) after 7 days. Colonies were selected and transferred to a new feeder layer with the same culture conditions from Day 30 to 50. Subsequently, hiPSC clones were passaged mechanically every 5–7 days, and the presumed undifferentiated regions were transferred to a new feeder layer. In our experiments, we used only undifferentiated colonies.

### Pharmacological experiments

Cultured neurons grown on coated coverslips or plates were exposed to increasing doses (10 and 100 μM) of ketamine for 1 h, 8 h, or 24 h to examine the effects of ketamine on the structural plasticity of the neurons. The medium was replaced at the end of the treatment period, and the neurons were transferred to an incubator and cultured for 48 h before subsequent morphological analyses. Other compounds (MK-801, CHIR-99021) were used at concentrations ranging from 1 to 100 μM (Supplementary Material I: Supplementary Table S1) and applied for at least 2 h. A pharmacological inhibitor was added to the cultures 30 min prior to treatments.

### Transfection of siRNA

Neurons were transfected with different concentrations of GSK-3β siRNA or scrambler siRNA as a control (Supplementary Material I: Supplementary Table S1). Then, the medium was replaced with a fresh medium after 48 h or 72 h. An immunofluorescence microscope detected the expression of GSK-3β in SPNs, and the appropriate dose of siRNA was selected for the following experiment. Ketamine exposure was performed 24 h after GSK-3β siRNA transfection.

### Lentivirus treatment

Neurons in a dish were transfected with HDAC6 shRNA lentivirus or scrambler lentivirus as controls at different MOIs (Supplementary Material I: Supplementary Table S1). The medium was then replaced with a fresh medium after 24 h. An immunofluorescence microscope detected the expression of HDAC6 in SPNs, and the appropriate dose of lentivirus was selected for the following experiments. Ketamine exposure was performed 48 h after HDAC6 shRNA lentiviral transfection.

### Immunofluorescence staining

Neurons were fixed with 4% ice-cold paraformaldehyde (PFA) in PBS for 20 min at room temperature (RT) and permeabilized with 0.2% Triton X for 20 min. Then, the neurons were blocked with 10% normal donkey serum and 0.1% Triton X for 1 h at RT. Incubation with primary and secondary antibodies (see Supplementary Material I: Supplementary Table S2) was performed overnight at 4 °C and 2 h at RT. Images were obtained using a Leica SP8 confocal microscope (Leica Microsystems, Japan). Fiji (ImageJ) software was used for cell counting and morphological analyses, which were performed to assess the growth cone, neurite length, neurite branches, and dendritic spine density from three coverslips per treatment group. Data were obtained from five fields for each culture condition.

### Transmission electron microscopy

The cells were fixed with 2% glutaraldehyde/2% PFA and postfixed with 1% OsO4 in 0.1 M phosphate buffer. The fixed cells were dehydrated in solutions containing increasing ethanol concentrations and then embedded in Durcupan resin at 6 °C. Thin sections of 70–80 nm were cut with a diamond knife on a Leica ultramicrotome and stained with uranyl acetate and lead citrate. The sections were imaged using a transmission electron microscope (Philips CM-120).

### Live SPN calcium imaging

Cal-520 calcium imaging of SPNs was performed as described in instructions. Day 60 neurospheres were attached to screening plates with glass bottom using Matrigel. Briefly, cells were loaded with 1 μM Cal-520 (Abcam, ab171868) for 60 minutes at 37 °C in HHBS medium, and then incubate the plate at room temperature for a further 30 minutes. The Cal-520 dye working solution was replaced with HHBS. Calcium transients were recorded using a 40X objective on a Leica TCS SP8 confocal microscope and at a frame rate of 1.35 Hz with 512 × 512-pixel resolution. Mean gray values were determined using Fiji software. Mean gray values were transformed to relative changes in fluorescence: dF/F(t) = (F(t) − F0)/F0, where F0 represents minimum gray values of the time series of each ROI. Calcium transients were detected as dF/F(t) crossed 3 median absolute deviation threshold.

### Western blotting

The protein extracts from differentiated cells were resolved on 12% SDS/PAGE gels, transferred to PVDF membranes, and incubated in a blocking solution with the indicated primary antibodies (see Supplementary Material I: Supplementary Table S2) at 4 °C overnight. Immunoreactive proteins were detected with horseradish peroxidase (HRP)-conjugated secondary antibodies and then visualized by a ChemiDoc XRS imaging system (Bio-Rad, Hercules, CA, USA). The immunoreactive bands were analyzed using plugins in Fiji (ImageJ) software. Representative Western blotting bands from three independent experiments are shown.

### Quantitative real-time PCR

According to the provider protocol, total RNA was extracted from hiPSC-derived striatal GABAergic neurons using the RNeasy Micro Kit (Qiagen, Germany). The quantity and purity of the RNA were assessed using a Nanodrop spectrophotometer. The RNA was reverse-transcribed using a First-Strand cDNA synthesis kit (Thermo Scientific, Carlsbad, CA, USA), and quantitative (q) PCR was performed using Fast Sybr Green PCR Master Mix. The primers used in this study were as follows: HDAC6 forward:5’-CGGAGGGTCCTTATCGTAGA-3’, reverse: 5’-GTAGCGGTGGATGGAGAAAT-3’; GSK-3β forward: 5’-AACACCAACAAGGGAGCAAA-3’, reverse: 5’-GAGCGTGAGGAGGGATAAGG-3’; GAPDH forward: 5’-TTGAGGTCAATGAAGGGGTC-3’, reverse: 5’-GAAGGTGAAGGTCGGAGTCA-3’. The ratio of the expression of the gene of interest and GAPDH as a housekeeping gene were calculated using the ΔΔCT method.

### Immunoprecipitation assay

After ketamine treatment, cells were lysed in ice-cold immunoprecipitation buffer and performed as previously described [31]. The lysate was incubated with rabbit anti-GSK-3β antibody overnight at 4 °C. ProteinA/G agarose (Beyotime Institute of Biotechnology) was added at 4 °C for 4 h. As a negative control, equivalent amounts of cell lysates were incubated with rabbit IgG (Beyotime Institute of Biotechnology). The mixture was finally detected by Western blotting.

### Statistical analysis

All statistical analyses were performed, and all graphical representations were produced using GraphPad Prism 7.0 software. The data are presented as the mean ± SEM unless otherwise specified. Structural plasticity parameter data (growth cone, neurite length, branches, and dendritic spine) and immunofluorescence data were normalized before measurement. Two groups were statistically compared using Student’s t test. Multiple groups were statistically compared with ordinary one-way or two way analysis of variance (ANOVA) followed by Tukey’s test for multiple comparisons. Each measurement was assessed at least in triplicate. *P* < 0.05 was considered significant.

## Results

### Characterizing ES/hiPSC derived SPNs

Striatal GABAergic SPNs originated from the progenitors of the lateral ganglionic eminences (LGE) [3]. We differentiated H9, an ES cell line, and hiPSC (8–12), a skin fibroblast-derived h line of19-age-old females, into forebrain ventral GABAergic neurons by supplying 200 ng/ml SHH from Days 10 to 25, based on the developmental trajectory of forebrain development (Supplementary Fig. 1A). Immunostaining with forebrain and striatal progenitor markers, including OTX2 and MASH1, showed that OTX2 + cells accounted for 90.3 ± 1.2% of cells, and MASH1 + cells accounted for 82.0 ± 2.7% of cells on Day 28 (Supplementary Fig. 1B, C). Growth cones of the SPN guide the striatal projections into the GPi and SNr in the fetal brain, forming the dominant descending longitudinal fiber systems in the forebrain [2]. Notably, the induced SPNs have the growth cones that expand and express MAP2 during maturation (Supplementary Fig. 1D). Using DARPP32 antibody and other GABAergic neuronal antibodies, including GABA, GAD65/67, MEIS2 and CTIP2, or panneuronal markers, including STEM121 and Tuj-1, we found that DARPP32 + cells accounted for 84.7.0 ± 3.2% of GABA + cells, 81.3 ± 2.0% of Tuj1+ cells, and 88.3 ± 2.9% of MEIS2 + cells; GABA + cells accounted for 90.7 ± 2.3% of STEM121 + cells; and GAD + cells accounted for 81.7 ± 4.0% of GABA + cells; and CTIP2 + cells accounted for 19.2% of DARPP32 + cells, revealing the relative purity of our SPN-inducing system (Supplementary Fig. 1E–G). On Day 50, the striatal GABAergic neurons displayed a more mature morphology with multiple medium-sized projections (Supplementary Fig. 1F). Immunostaining with antibodies against the synapse markers Bassoon or PSD95 and GABA showed that GABAergic neurons presented synapses (Supplementary Fig. 1H, I). The induced neurons from the hiPSC (8–12) culture over 90 days exhibited spontaneous action potentials (Supplementary Fig. 1J). Considering these results, our induced SPNs is suitable for studying the effects of ketamine on the development of SPNs.

### Ketamine impaired growth cone expansion in early immature SPNs

**Fig. 1:**
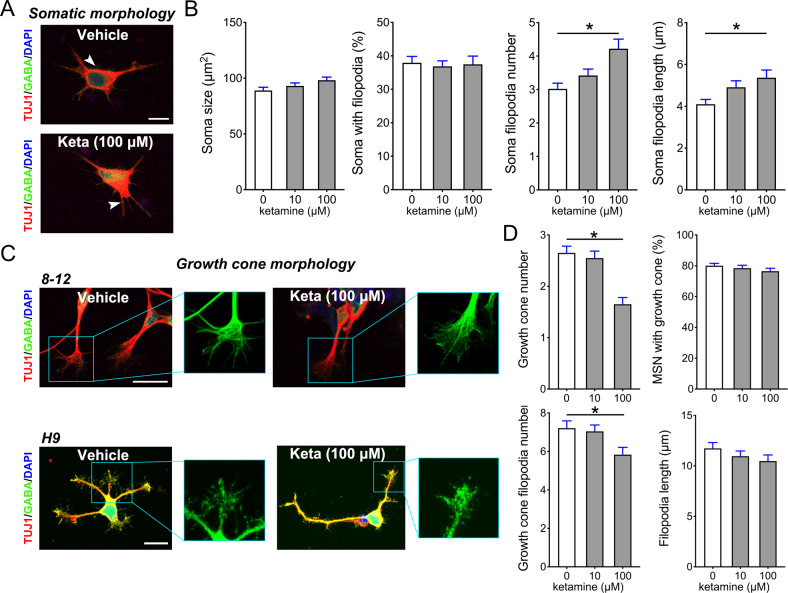
Ketamine exposure impairs immature SPN growth cones. **A** Representative Tuj1 and GABA antibodies immunostained filopodia-like extensions of ketamine-treated striatal projections neurons (SPNs) and control SPNs on Day 26. White arrow, short or long protuberances in soma. Scale bar, 10 μm. **B** Comparison of the soma size (*n* = 60), percentage of cells with filopodia (*n* = 15), soma filopodia (including long and short protuberances) number (*n* = 50) and length (*n* = 23) in 10 µM, 100 µM ketamine treated SPNs with control SPNs. **C** Representative Tuj1 and GABA antibodies immunostained growth cones in the ketamine-treated SPNs and vehicle SPNs on Day 26 (the boxed region, magnified growth cone in the right panel). SPNs are derived from H9 and hiPSC (8–12). Scale bar, 25 μm. **D** The growth cone counts (*n* = 20), growth cone ratio (*n* = 15), filopodia number (*n* = 25) and length (*n* = 25) of SPNs in the 10 and 100 µM ketamine-treated groups were compared with those in the control group. Data of SPNs derived from H9 and hiPSC (8–12). Data, mean ± s.e.m. One way ANOVA. *P < 0.05.

Based on our previous work [18], we further tested the effect of ketamine on the growth cone, including lamellipodium and filopodia expansion in induced GABAergic neurons at the earlier period of the induction-immature period. We exposed the differentiated GABAergic neurons to 10 or 100 μM ketamine at Day 26 for 1 h, and stained them with Tuj-1, a pan-neuronal marker, and GABA antibodies. Here, we here only report partly significant results, for summary statistics see Supplementary Material II: Table S3. The results of Tuj-1 and GABA staining revealed that ketamine did not affect neuronal soma size or filopodia, whereas increased the count and length of thin filopodia protruding from the soma (F1 = 5.190, P1 < 0.001 and F2 = 4.401, P2 = 0.016) (Fig. 1A, B); 24 h of ketamine exposure rendered the growth cones collapse, manifested by the shrinking of growth cone lamellipodium and filopodia; the effects of 100 μM ketamine on growth cone lamellipodium and filopodia were more significant than those of other dosages (F1 = 17.250, P1 < 0.001 and F2 = 4.234, P2 = 0.018) (Fig. 1C, D). These findings revealed that a higher dosage of ketamine exposure also retarded or impaired growth cone lamellipodium and filopodia expansion.

### Ketamine exposure caused neurite retraction and aberrant synaptogenesis in SPNs at the mature stage

**Fig. 2:**
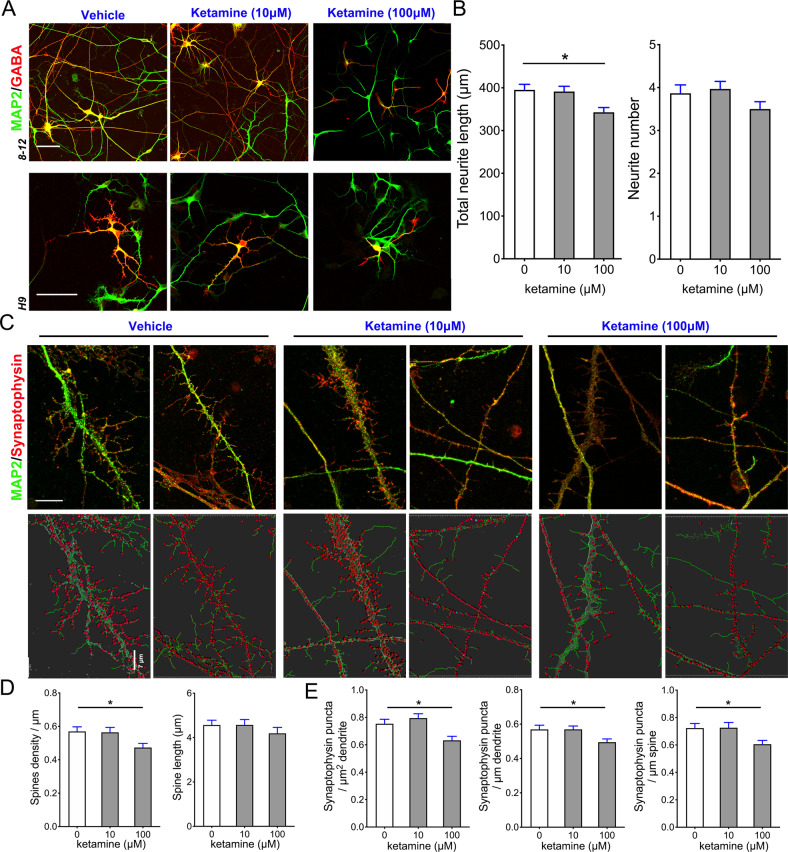
Ketamine impairs dendrites and synapse of mature SPNs. **A** Comparison of the dendrites of SPNs in the vehicle and ketamine groups after 24 h of exposure. Scale bar, 50 μm. **B** Comparison of the neurite length (*n* = 22) and neurite number (*n* = 30) of SPNs in the vehicle and ketamine groups after 24 h of exposure. **C** Representative MAP2 and synaptophysin antibody-immunostained dendrites of the vehicle and ketamine-treated SPNs on Day 45. The lower panel is the 3D rendered structure, and it showed synaptophysin+ puncta on the dendrite and spine. Scale bar, 10 μm. **D**, **E** Spine density (*n* = 18) and length (*n* = 23) and synaptic (computed synapse density from dendrites and spine) parameters (*n* = 15, 21 and 23) were compared between the SPNs treated with ketamine and the control. Data are from H9 and hiPSC derived SPNs. Data, mean ± s.e.m. One way ANOVA. *P < 0.05.

**Fig. 3:**
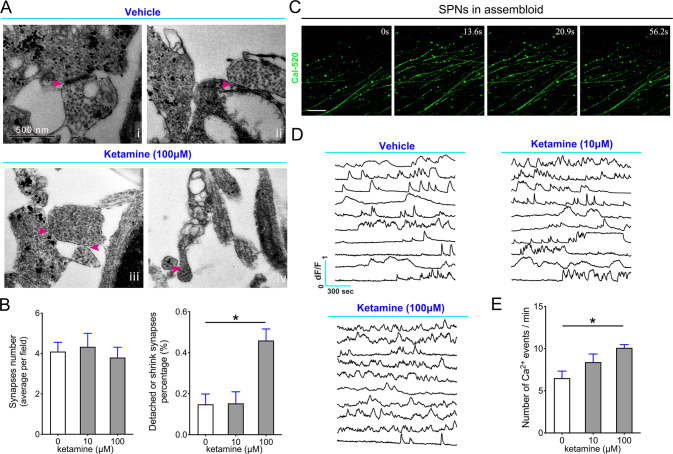
Ketamine impairs the synapse and disrupts calcium activities in SPNs. **A** Representative TEM images of the vehicle and ketamine-treated SPNs on Day 50. Magenta arrowhead: synapse and synaptic vesicles. Data are H9 derived SPNs. Scale bar, 500 nm. **B** Comparison of the synaptic ultrastructural changes in TEM images of ketamine treated SPNs and control SPNs (*n* = 10). Scale bar, 25 μm. **C** Representative time-lapse images of calcium activities in SPNs after ketamine treatments. **D**, **E** Traces showing calcium activity of corresponding ROIs in the control, 10 µM and 100 µM groups revealed the intensification of calcium activities in the SPNs of the 100 µM group (*n* = 10). Data, mean ± s.e.m. One way ANOVA. *P < 0.05.

In our induction protocol, induced GABAergic neurons in the dishes expressed DARPP32, a molecular signature for GABAergic neuron maturation around Day 45 [32]. To determine whether ketamine exposure affects mature SPNs in dishes, we exposed induced GABAergic neurons at 45 days with 10 or 100 μM ketamine for 24 h and assessed neurite morphology and synaptogenesis in SPNs on Day 47 and Day 50. We found that untreated neurons elaborated complex neurites during culture, whereas a single dosage of ketamine treatment disrupted SPNs neurite outgrowth (F = 5.661, P = 0.006) (Fig. 2A, B). Using immunostaining with MAP2 antibody, a dendritic marker, we observed that 100 μM ketamine decreased the dendritic spine count (F = 3.823, P = 0.029) but not the spine length on primary dendrites (Fig. 2C, D). Using synaptophysin immunostaining, we found that ketamine significantly reduced the synaptic count (F1 = 7.300, P1 = 0.002; F2 = 4.137, P2 = 0.020 and F3 = 4.346, P3 = 0.017) (Fig. 2E), paralleling the reduction in dendritic arborization and spine formation on Day 50. To observe the structural changes in the synapse after ketamine exposure, we used TEM to observe the ultrastructurally changes of SPNs synaptic structure after 10 and 100 μM ketamine treatments for 45 days. We observed multiple synapses with abundant synaptic vesicles in untreated or 10 μM ketamine treated GABAergic neurons, whereas the presynaptic membrane detachments and dwindling presynaptic parts were observed in the 100 μM ketamine-treated group (F = 11.12, P < 0.001) (Fig. 3A, B). We also detected the physiological activity of ketamine-treated SPNs by calcium imaging (Fig. 3C, D) and found that 100 μM ketamine treatment increased Ca^2+^ activity in SPNs (F = 5.480, P = 0.010) (Fig. 3E). These results revealed that 100 μM ketamine treatment not only collapsed growth cone and filopodia expansion at the early stage (immature) in GABAergic neurons but also reduced synapse formation and disrupted the existing synapses of mature GABAergic neurons.

### GSK-3β activation contributes to the ketamine-induced reduction of synaptogenesis and growth cone collapse in GABAergic neurons

GSK-3β and α-tubulin staining signals coexisted and partially overlapped in the growth cones and neurites at immature and mature stages (Fig. S2A). This observation implied that the kinase activity of GSK-3β might regulate α-tubulin. Phosphorylation of GSK-3β at serine 9 inhibits GSK-3β activity [33]. Thus we used a p-GSK-3β (ser9) antibody to evaluate GSK-3β activation levels and found that ketamine treatment increased GSK-3β levels while decreasing p-GSK-3β (ser9) levels (t1 = 4.296, P1 = 0.013 and t2 = 6.467, P2 = 0.003) (Supplementary Fig. 2B–D). These results indicate that activation of GSK-3β by suppressing p-GSK-3β (ser9) contributes to ketamine-mediated growth cone collapse and synaptic reductions in GABAergic neurons.

**Fig. 4:**
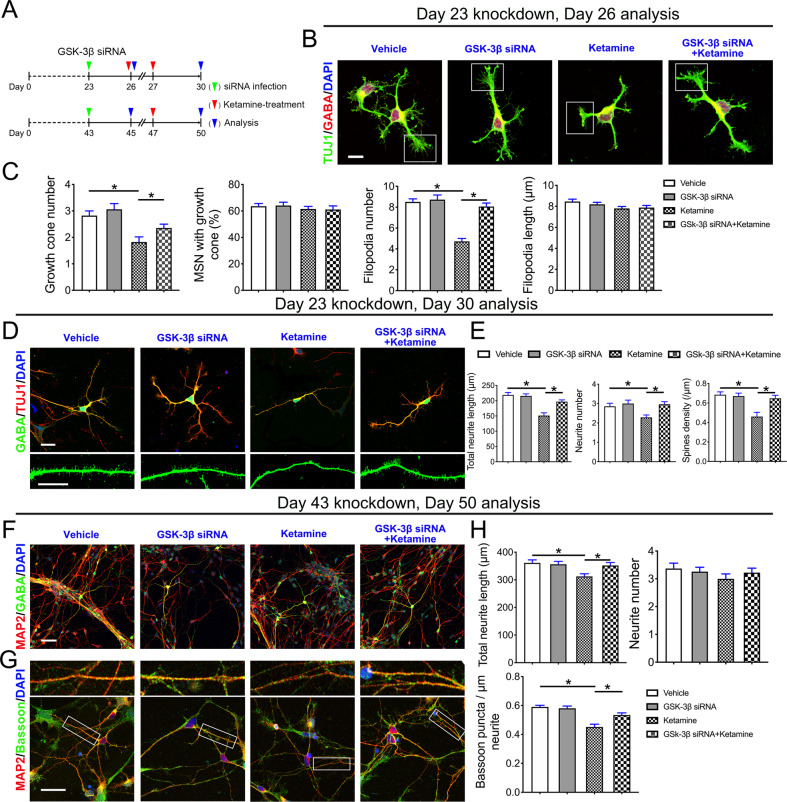
Downregulation of GSK-3β alleviates the effect of ketamine on developing SPNs. **A** Schematic of *GSK-3β* knockdown experiments. **B** Representative images of SPNs in the vehicle, GSK-3β siRNA, and ketamine-treated groups on Day 26. The boxed region shows each group’s the typical morphology of representative growth cone. Scale bar, 10 μm. **C** Comparison of the growth cone number (*n* = 17), percentage of cells with growth cones (*n* = 10), filopodium number (*n* = 18) and length (*n* = 49) in the SPNs of the GSK-3β siRNA group with vehicle and ketamine-treated groups. **D** Representative dendrites of the vehicle-, GSK-3β-siRNA- and ketamine-treated SPNs on Day 30. Scale bar, 25 μm. **E** Comparison of the total neurite length (*n* = 21), number (*n* = 28), and spine density (*n* = 14) of SPNs in the GSK-3β-siRNA group and ketamine-treated group. **F**, **G** MAP2/GABA and MAP2/Bassoon immunostained images of the vehicle group with drugs and the ketamine-treated group at Day 50. Scale bar, 50 μm. **H** Comparison of the total neurite length (*n* = 29), neurite number (*n* = 27), and synaptic (computed synapse density from dendrites) parameters (*n* = 45) of SPNs in the vehicle group with drugs or ketamine-treated group at Day 50. Data, mean ± s.e.m. One way ANOVA. *P < 0.05.

To test whether the GSK-3β pathway contributes to the ketamine-induced kinetic impairments of neurite arborization in SPNs, we performed small interfering RNA (siRNA)-mediated GSK-3β knockdown experiments (t = 3.238, P = 0.032) (Supplementary Fig. 2E–G). After GSK-3β siRNA transfection, the neurons were exposed to ketamine for 1 or 24 h and analyzed after another 12–48 h of culture (Fig. 4A). We found that the reduction of GSK-3β with siRNA transfection alone did not alter neuronal morphometry, whereas the reduction of GSK-3β effectively prevented ketamine-induced growth cone collapse (F1 = 8.598, P1 < 0.001 and F 2 = 29.6, P2 < 0.001) (Fig. 4B, C). Reducing GSK-3β expression promoted neurite outgrowth and spine density (F1 = 16.830, P1 < 0.001; F2 = 4.922, P2 = 0.003 and F3 = 9.334, P3 < 0.001) (Fig. 4D, E). Furthermore, the downregulation of GSK-3β also ameliorated neurite retraction and aberrant synaptogenesis induced by a higher dosage of ketamine (F1 = 4.629, P1 = 0.004 and F2 = 16.850, P2 < 0.001) (Fig. 4F–H).

**Fig. 5:**
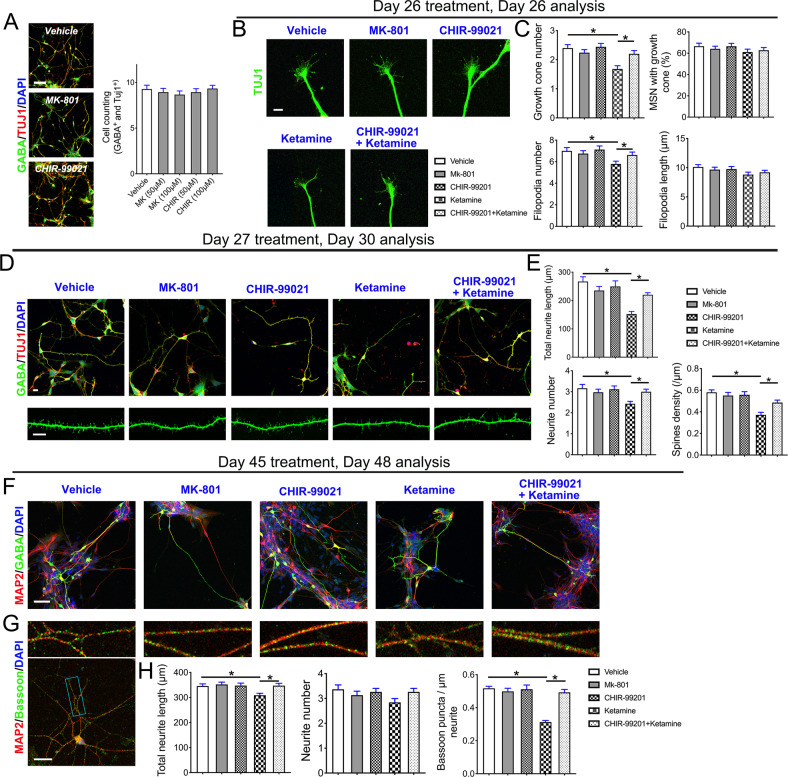
Ketamine impairs the morphological maturation of developing SPNs by activating the GSK-3β pathway. **A** SPNs were treated with the labeled drugs on Day 26 and analyzed after treating for 4 h (drug concentrations: MK-801, 50 and 100 μM; CHIR-99021, 50 and 100 μM). Representative images and ratio of SPN counts/total cells identified by nucleus (DAPI staining) (*n* = 15). **B** Representative images of SPNs with expanded growth cones costained by GABA and Tuj1 antibodies in the vehicle and drugs- or ketamine-treated groups. Scale bar, 10 μm. **C** The growth cone number (*n* = 25), percentage of cells with growth cones (*n* = 10), filopodium number (*n* = 24), and length (*n* = 25) of SPNs in the vehicle with drugs and ketamine-treated groups were compared. **D** Dendritic morphology in the vehicle with drugs SPNs and ketamine-treated SPNs on Day 30. Scale bar, 50 μm. **E** Calibration of total neurite length (*n* = 30), neurite number (*n* = 42) and spine density in the vehicle and drugs- or ketamine-treated groups (*n* = 16). MAP2/GABA (**F**) and MAP2/Bassoon immunostaining (**G**) images of the vehicle and drugs- or ketamine-treated neurons at Day 45. Scale bar, 50 μm. **H** Calibration of total neurite length (*n* = 39), neurite number (*n* = 38), and synaptic (computed synapse density from dendrites) parameters (*n* = 31). Data, mean ± s.e.m. One way ANOVA. *P < 0.05.

A previous study reported GSK-3β proteins have key roles in many fundamental processes during neurodevelopmental [33]. To determine the status of GSK-3β pathways in ketamine-induced growth cone collapse and synaptic reduction in GABAergic neurons, we treated differentiated GABAergic neurons with MK-801, an NMDAR inhibitor [34], or CHIR-99021, a GSK-3β inhibitor [35], and tested whether the two drugs replicated or reversed ketamine-induced growth cone collapse and synaptic reduction in immature or mature GABAergic neurons. By evaluating nuclear morphology and cellular detachments, we found that both 50 and 100 μM MK-801 and CHIR-99021 did not induce wide apoptosis in the neurons characterized by nuclear fragmentation or cellular detachments from culturing slides (Fig. 5A). We found that neither MK-801 nor CHIR-99021 completely replicated the ketamine-mediated growth cone collapse or synaptic reduction in SPNs (Fig. 5B–H). However, CHIR-99021 pretreatment alleviated ketamine-induced growth cone collapse (F1 = 7.258, P1 < 0.001 and F2 = 3.275, P2 = 0.014), neurite length shortening, and spinal reduction (F1 = 9.666, P1 < 0.001; F2 = 4.104, P2 = 0.003 and F3 = 11.060, P3 < 0.001) (Fig. 5B–E). In addition, CHIR-99021 pretreatment also rescued ketamine-induced neurite retraction and aberrant Bassoon expression (F1 = 3.840, P1 = 0.005 and F2 = 23.490, P2 < 0.001) (Fig. 5F–H). These results suggest that ketamine impairs morphological maturation of developing SPNs by activating GSK-3β.

### Ketamine exposure enhanced the interaction of GSK-3β with HDAC6 in GABAergic neurons

**Fig. 6:**
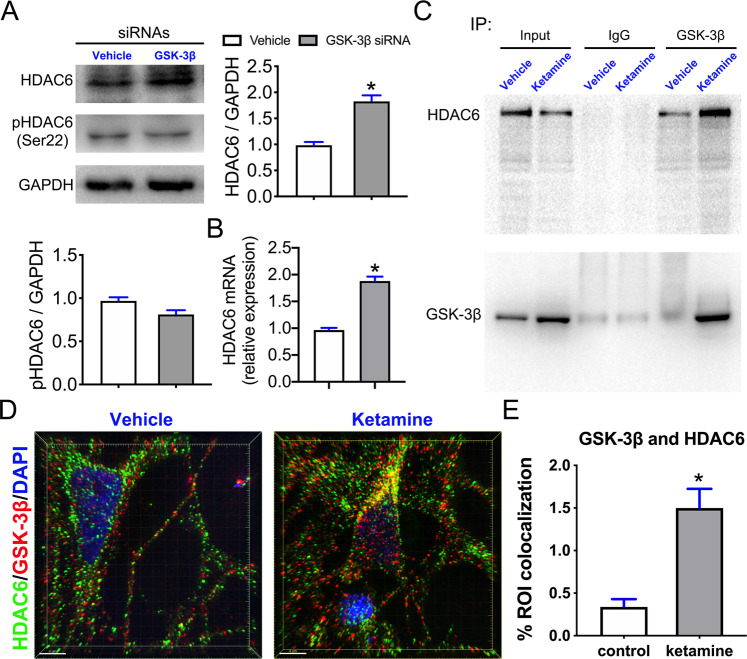
Ketamine enhanced the direct interaction of GSK-3β with HDAC6. **A** The immunoblotting results of HDAC6 and pHDAC6 (ser22) antibodies in SPNs cotransfected with GSK-3β knockdown siRNA and the measurement of protein levels (*n* = 3). **B** Testing *HDAC6* transcription after GSK-3β knockdown siRNA transfection by qRT-PCR (*n* = 3). **C** Co-IP of GSK-3β and HDAC6 in the vehicle and ketamine-treated SPNs showed that ketamine treatment enhanced the direct interaction. **D** Representative high resolution images with z-stacks of GSK-3β and HDAC6 immunostaining taken by SIM microscopy in the vehicle and ketamine-treated SPNs. Scale bar, 4 μm**. E** Comparison of GSK-3β- and HDAC6-positive values of ROI in the SPNs of the vehicle- and ketamine-treated group (*n* = 5). Data, mean ± s.e.m. Student’s *t* test. *P < 0.05.

We found that knockdown of GSK-3β in SPNs increased the expression and transcription of HDAC6 (t1 = 6.407, P1 = 0.003 and t2 = 9.912, P2 < 0.001), whereas it did not affect p-HDAC6 (ser 22) levels (Fig. 6A, B). To determine whether GSK-3β directly regulates HDAC6, we performed coimmunoprecipitation (Co-IP) and found that HDAC6 directly bind with GSK 3β (Fig. 6C). The data showed that ketamine exposure dramatically enhanced the interaction between GSK-3β and HDAC6 (Fig. 6C), and immunostaining images of GSK-3β and HDAC6 obtained with SIM microscopy demonstrated that ketamine exposure dramatically increased the colocalization of HDAC6 and GSK-3β (t = 4.782, P = 0.001) (Fig. 6D, E). These data suggest that HDAC6 is a direct downstream target of GSK-3β during ketamine exposure in developing GABAergic neurons.

### Inhibiting HDAC6 partially replicates the effect of ketamine exposure on the growth cone and synaptogenesis in SPNs

Previously, we observed that ketamine decreased HDAC6 expression and HDAC6 kinase inhibitors inhibited dendrite branching and dendritic spine formation in GABAergic neurons [18], implying that growth cone expansion and synaptogenesis might also depend on HDAC6. Thus, we detected the changes in HDAC6 transcription levels in induced GABAergic neurons and found that HDAC6 transcription levels increased from Day 20 to 35, and the HDAC6 transcription levels on Days 35, 40, and 45 were nearly two times higher than those on Day 20 (Supplementary Fig. 3A). Furthermore, we observed that HDAC6 was distributed along dendritic clusters with α-tubulin (Supplementary Fig. 3B–D), which impacts the kinetics of the cytoskeletal [36]. These findings indicated that transcriptional regulation of HDAC6 is crucial for GABAergic neuronal growth cone expansion and synaptogenesis.

**Fig. 7:**
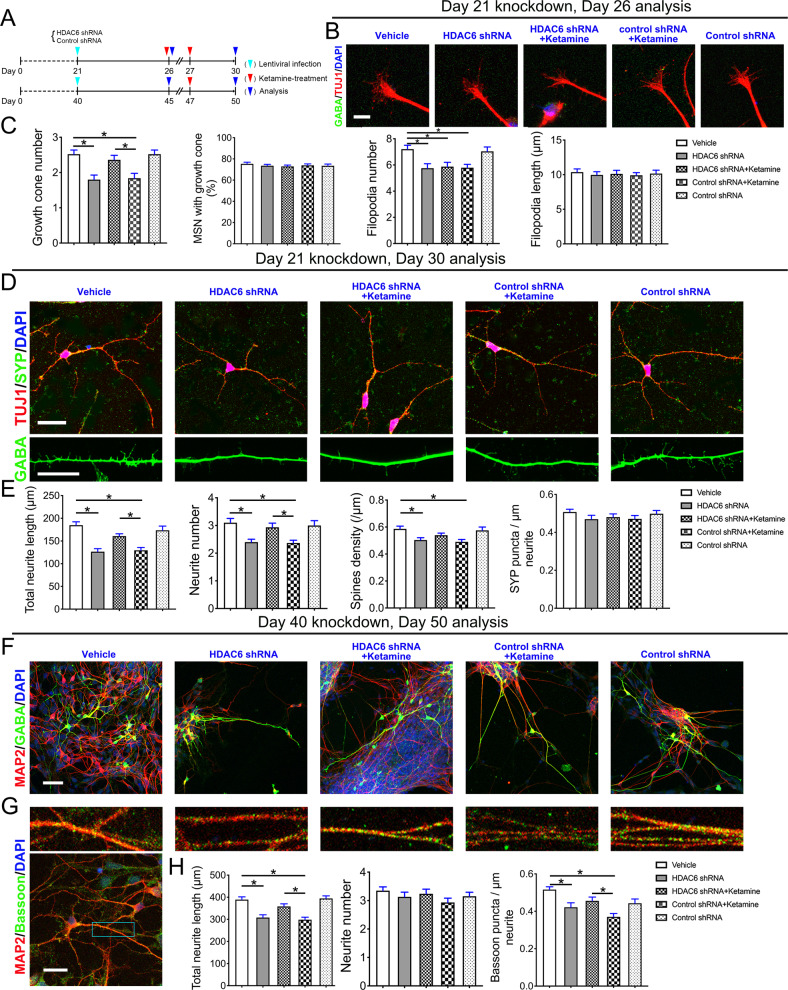
Ketamine impairs the morphology and maturation of developing SPNs by inhibiting HDAC6. **A** Schematic of the experiments. Neurons were coinfected with a lentivirus downregulating HDAC6. **B**, **C** Representative growth cone images of SPNs in the vehicle, HDAC6 shRNA, HDAC6 shRNA + ketamine, control shRNA + ketamine and control shRNA groups on Day 26. Scale bar, 10 μm. Calibration of growth cone number (*n* = 25), percentage of cells with growth cones (*n* = 20), filopodium number (*n* = 24), and length (*n* = 20) of SPNs in the vehicle, HDAC6 shRNA, HDAC6 shRNA + ketamine, control shRNA + ketamine, control shRNA groups. **D**, **E** Representative neuronal of dendritic morphology of SPNs in the vehicle, HDAC6 shRNA, HDAC6 shRNA + ketamine, control shRNA + ketamine, control shRNA groups on Day 30 Scale bar, 25 μm. Calibration of total neurite length (*n* = 30), neurite number (*n* = 30) and spine density (*n* = 21) of SPNs in each group. Representative MAP2/GABA (**F**) and MAP2/Bassoon (**G**) immunostained SPNs in in vehicle, HDAC6 shRNA, HDAC6 shRNA + ketamine, Control shRNA + ketamine, and control shRNA groups on Day 50. Scale bar, 50 μm. **H** Calibration of total neurite length (*n* = 36), neurite number (*n* = 46), and synaptic (computed synapse density from dendrites) parameters of SPNs (*n* = 24) in each group. Data, mean ± s.e.m. One way ANOVA. *P < 0.05.

Then, we inhibited HDAC6 with short hairpin RNA [shRNA] lentiviruses and observed that inhibiting HDAC6 produces the same effect as ketamine on the growth cone and synaptogenesis (Supplementary Fig. 4). By calibrating the growth cone exposed to ketamine on Day 26, Day 27, and Day 47 (Fig. 7A), we found that inhibiting HDAC6 on Day 21 resulted in growth cone collapse (F1 = 8.188, P1 < 0.001 and F2 = 5.395, P2 < 0.001) (Fig. 7B), resembling the effect of ketamine exposure on the growth cone and synaptogenesis on GABAergic neurons. However, inhibiting HDAC6 did not reverse the ketamine-induced reduction in the number of growth cone filopodia (Fig. 7C). Inhibition of HDAC6 transcription also strongly reduced dendritic arborization and the number of dendritic spines (F1 = 13.710, P1 < 0.001; F2 = 6.257, P2 < 0.001 and F3 = 4.860, P3 = 0.001) **(**Fig. 7D, E), which partially replicated the effects of ketamine and HDAC6 kinase inhibitors. The decrease in *HDAC6* transcription could partially but significantly rescued the ketamine-induced complete blockade of neurite outgrowth (F1 = 13.710, P1 < 0.001 and F2 = 6.257, P2 < 0.001) (Fig. 7E). The decrease in *HDAC6* transcription on Day 40 mildly to moderately reduced neurite length and reduced the number of synapses compared to ketamine treatment (F1 = 14.130, P1 < 0.001 and F2 = 6.734, P2 < 0.001) (Fig. 7F–H). The decrease in *HDAC6* transcription completely rescued the ketamine-mediated defects in dendritic morphology and synapse numbers (F1 = 14.130, P1 < 0.001 and F2 = 6.734, P2 < 0.001) (Fig. 7H). These results suggest that inhibiting HDAC6 replicates, to a significant extent, growth cone collapse and synaptic reduction caused by ketamine exposure during neurodevelopment. Inhibiting HDAC6 partially rescued ketamine-induced growth cone damage and synaptic reduction. These results are consistent with the notion that ketamine’s effect on developing neurons is caused by cytoskeletal dysregulation and that ketamine affects neuronal development by decreasing HDAC6 transcription.

## Discussion

The striatum, which originated from the LGE, wires complicated and precise projection and synaptic networks with the substantia nigra and the pallidum in the midbrain [37–39]. The mature striatum comprises 95% SPNs. The functional and structural disruptions in striatal SPNs, including neuronal death and aberrant projections and connections, cause a wide range of brain diseases, such as schizophrenia, ASD, Gilles de la Tourette syndrome, OCD and OC-spectrum disorders and HD [5–12]. Ketamine, a safe and widely used anesthetic with a lower dose in pediatrics, has a detrimental effect on adult neurons at higher doses. Despite reports that therapeutic or subanesthetic dosages improve abnormal projections in the adult murine brain [40–42], abundant data in humans, primates, and rodents indicate that chronic and higher dosage ketamine exposure has lasting and irreversible effects on brain functions, including the disruptions of neuronal connections and wiring. For example, ketamine reduced the frontotemporal and temporoparietal functional connectivity in humans [13]; chronic ketamine irreversibly damaged brain functions in primates [14]; and ketamine exposure increased anxiety, depression-like behavior, and cognitive impairment in progeny after maternal exposure [15], and crippled fetal and juvenile brain development and functions [16, 17]. Our recent data from a fused human cortical and the striatal organoid system showed that 100 μM ketamine for 24 h significantly decreased the subcortical projection of human cortical organoids that targeted human striatal organoids (unpublished data). Inducible GABAergic neurons in a dish mimic the developmental trajectory of GABAergic neurons in vivo and provide a model to test the toxicity of the ketamine effect [32]. We tested the ketamine dosage-dependent effect on GABAergic growth and maturation using this system. We found that exposure to 100 μM ketamine caused growth cone collapse and reduced synaptogenesis, dendrite lengths and count, and physiological activities, and caused the detachment of synapse by deregulating HDAC6- and GSK-3β-dependent pathways. Ketamine effects are dominant in early development but are alleviated in mature neurons. These findings might partially address the effects of ketamine on the behavior changes in fetuses and adults.

The growth cone is a motile structure that is present at the tip of synapse extension and mediates the neuronal protrusion growth [43]. Motile growth cones present a unique extension morphology (lamellipodium and filamentous pseudopods), and the repulsive molecules on the posterior surface of adjacent cells change growth cone morphometry [44], regulating the direction of neuronal extension. However, the extensive disruption of growth cones in growing or regenerating neural tissue by external substances (e.g., anesthetic [45]) may interfere with the typical cellular structure establishment in the developing nervous system. Ketamine exposure in immature GABAergic neurons reduced lamellipodium or filamentous pseudopods of growth cones hindering the later growth and extension of neurites in SPNs. Ketamine also impaired the dendrites of neurons on Day 30, presenting a later development to a lesser extent on Day 45. Consistent with the shortening of dendrites and growth cone retardation, ketamine exposure decreased synapses density on mature neurons.

The activity of Ca^2+^ parallels the structural and functional alterations in the neurons caused by ketamine [46]. 1–10 μM ketamine could increase the amplitude of Ca^2+^ peaks [47], but 100–200 μM ketamine reduce the amplitude of Ca2+ peaks in hippocampal neurons [48]. Recently, synaptic Ca^2+^ transients and deficient inhibitory mechanisms produced by subanesthetic ketamine were demonstrated to retard the growth of dendritic spines during neuronal development [49]. Furthermore, Ali and colleagues reported that ketamine acutely suppressed the activity of somatostatin-expressing (SST) interneurons in the medial prefrontal cortex of adult mice, leading to greater synaptically evoked calcium transients in the apical dendritic spines of pyramidal neurons [50]. Our study found 100 μM ketamine raised calcium activity in SPNs, accompanied by decreased synapse density in mature neurons. These results suggest that the effects of ketamine on synaptic plasticity and neuronal response may be dependent on age and specific brain regions.

Ketamine regulated neuronal development by NMDA receptor inhibition, GSK-3β signaling pathway activation, brain-derived neurotrophic factor expression changes, etc. [51, 52]. We found that inhibition of NMDA receptors alone does not affect growth cones, filopodia, branches of neurons, or dendritic spines in SPNs. Similarly, the inhibition of NMDA receptors during neuronal maturation did not change the expression of neuronal or synapse-related proteins. Previous studies have proposed that the mechanism of NMDA antagonists is to produce neurotoxicity by releasing the inhibitory control of excitatory input to rat neurons [53, 54]. NMDA receptor antagonists did not show the rapid, effective, and long-lasting antidepressant effect that resembles ketamine [55]. Thus, ketamine effects that are independent of NMDA receptors have become a focus of research [56]. Here, we found that ketamine increased the activity of GSK-3β. Consistent with our observation, decreased phosphorylation of GSK-3β (Ser9) led to shrinking dendrites [25, 57]. The aberrant activation of GSK-3β related to neurological and psychiatric disorders characterized developmental abnormalities and altered neurocircuitry stability [58, 59]. The dysregulation of GSK-3β in various brain abnormalities supports that GSK-3β is a critical player in neuronal functions, from brain bioenergetics to the establishment of neuronal circuits, the modulation of neuronal polarity, migration, and proliferation [60, 61]. In particular, GSK-3β was shown to have a role in the phosphorylation of cytoskeletal proteins, which impacts neuronal plasticity [62]. Cytoskeletal components that regulate the development and maintenance of neurites and stabilize/destabilize microtubules influence the structure of dendrites, spines, axons, and synapses. This finding is consistent with other work on GSK-3β inhibitors [63, 64]. We found that CHIR-99021 improved or restored the neuronal structure and synaptic maturation in SPNs. The extension of neurites and synapses of human GABAergic neurons was completely restored when the cells were treated with CHIR-99021. Similarly, the downregulation of HDAC6 alleviated ketamine-induced inhibition of growth cone, neurite, and dendritic spine development, reversed neuronal synapse damage during the maturation process, and played a particular protective role.

HDAC6 traffics neurotrophic factors, acts as α-tubulin deacetylase, and transports mitochondria in hippocampal neurons [36, 65]. HDAC6 dysfunction led to hyperacetylation of α-tubulin and retarded axonal and dendritic growth in the hippocampus [66–68]. Previously, we found that ketamine reduced HDAC6 and hyperacetylated α-tubulin in SPNs [18]. Inhibiting or knockdown of HDAC6 replicated ketamine-induced dendritic dysgenesis and synapse loss, and HDAC6 deletion partially rescued ketamine-induced impairments. Growth cone extension is first driven by filamentous actin (F-actin) polymerization [43, 44], while dendrite outgrowth, and synapse formation requires a deacetylase to remove acetyl groups from tubulin to destabilize microtubules [1, 69]. This may explain how down regulation of HDAC6 led to different outcome of ketamine-induced growth cone filopodia, dendritic morphology and synapse numbers. HDAC6 deletion on Day 21 failed to alleviate growth cone collapse, but neurite branching and synapse formation were significantly reversed after HDAC6 knockdown on Day 30, which in line with reports that HDAC6 inhibition can restore nerve conduction [70, 71]. In addition, HDAC6 inhibition can protect against neurotoxic insults in various neurodegenerative disease models [72, 73]. Based on our observation, inhibiting HDAC6 by ketamine might be one of the dominant ways ketamine affects the fetal brain. HDAC6 activity depends on direct or indirect binding of related proteins that cause phosphorylation, and GSK-3β binds HDAC6, enhancing HDAC6 activity [74, 75]. GSK-3β may activate HDAC6 through phosphorylation of the serine (Ser-22) site, leading to deacetylation of α-tubulin and breakdown of microtubule structure [76]. In contrast, inhibition of GSK-3β activity can reduce HDAC6-mediated tubulin acetylation [77]. We found that GSK-3β is an upstream molecule of HDAC6 expression, and ketamine treatment increased the interaction of HDAC6 without changing HDAC6 phosphorylation levels, indicating that other kinases but not GSK-3β phosphorylated HDAC6 in neurons.

We showed that ketamine affects the neuronal structure of early developmental and mature striatal GABAergic neurons. Ketamine exposure in the early stage of neuronal development leads to massive defects in dendritogenesis and synaptogenesis through aberrant activation of GSK-3β and HDAC6-mediated hyperacetylation of α-tubulin. The current study demonstrates that altering microtubule acetylation benefits neuronal structural development in ketamine exposure.

## Supplementary information

Supplementary Material I

Supplementary Material II

Supplementary Figure 1

Supplementary Figure 2

Supplementary Figure 3

Supplementary Figure 4
