# Supplementary material for: Ketamine impairs growth cone and synaptogenesis in human GABAergic projection neurons via GSK-3β and HDAC6 signaling
Source: Mol Psychiatry. 2022 Nov 21;29(6):1647–59. doi: 10.1038/s41380-022-01864-5 (PMC11371642; doi:10.1038/s41380-022-01864-5)
Supplement: Supplementary file 1 — Supplementary Material I [file 41380_2022_1864_MOESM1_ESM.doc]

**Supplementary Figure Legends**

**Supplementary Figure 1. Characterizing of hiPSC**

**A.** The differentiating strategy of hiPSC into GABAergic projection neurons. **B.** The immunostaining results of OTX2 and MASH1 antibodies at Day 28. Most neurons labeled by OTX2 and MASH1 antibodies. Scale bar, 100 µm. **C.** Counts of OTX2 and MASH1+ neurons in dishes (n=3). **D.** Tuj1 and GABA antibody-immunostaining showed smaller growth cones on projections in SPNs with several projections on Day 30 Scale bar, 5 µm. **E.** DARPP32 and MAP2 antibodies immunostainings revealed the cytoskeleton of expanded growth cone in SPNs on Day 50. Scale bar, 2 µm. **F.** The immunostainings results of combining neuronal markers including Tuj1 and STEM121 with forebrain markers including MEIS2, GABA、DARPP32, GAD, and CTIP2 in SPNs derived from hiPSC (8-12). Induced neurons at 50 days present a mature morphology with more projections and branches (GAD and GABA immunostaining) Scale bar, 50 µm. **G.** The percentage of SPNs in the culturing dishes (n=3). **H, I.** The images of Bassoon and GABA antibody-immunostaining taken by SIM microscopy with near 64 nm resolution showed multiple synapses on the longer projections of GABAergic neurons on Day 80. Scale bar, 20 µm. J. The action potentials of SPNs that were differentiated for over 100 days by whole-cell patch clamping (hiPSC, 8-12). Data, mean ± SD.

**Supplementary Figure 2. GSK-3β expression in the developing SPNs after ketamine treatment and its siRNA identification**

**A.** Representative images of Day 26, 30 and 45 SPNs immunostained for GSK-3β and α-tubulin. White arrow, growth cone. Scale bar, 5 μm (Day26) and 25 μm (Day 30 and 45). **B.** The changes of GSK-3β and pGSK-3β (ser9) levels by immunoblotting in SPNs after 100 µM ketamine treatment for 24 h on Day 30. **C.** Measuring immunoblotting of GSK-3β and pGSK-3β (ser9) levels in SPNs (*n*=3). D. Quantitative real-time PCR analysis of GSK-3β expression after ketamine treatment (*n*=3). **E.** Representative images of neurons co-transfected with GSK-3β siRNA at the concentration of 0, 20, 50 and 100 nM. Scale bar, 25 μm. **F, G.** Western blot (F) and RT-PCR (G) analyses of GSK-3β expression were performed after GSK-3β siRNA transfection (*n*=3). Data, mean ± s.e.m. Student’s t -test. *, *P*<0.05.

**Supplementary Figure 3. HDAC6 expression in developing SPNs**

**A.** Quantitative RT-PCR for HDAC6 mRNA in SPNs on Day 20, 25, 30, 35, 40 and 45 (*n*=3). Data, mean ± s.e.m. **B.** Representative images of the growth cones of SPN on Day 26 immunostained for Tuj1 (rectangle) or ɑ-tubulin (square) and HDAC6 (white arrowhead, growth cone). HDAC6 stained puncta in the growth cone paralleled with α-tubulin. Scale bar, 5 μm. **C, D.** Representative images of day 30 and 45 SPNs immunostained by HDAC6 and α-tubulin (C), or MAP2 or α-tubulin antibodies (D). Scale bar, 25 μm.

**Supplementary Figure 4. HDAC6 shRNA lentiviral identification in SPNs**

**A.** Representative images of SPNs transfected with HDAC6 shRNA lentivirus at the MOI of 1, 10, 50 and 100. Scale bar, 25 μm. **B.** Immunoblotting of HDAC6 antibody in SPNs transfected with HDAC6 or control shRNA lentivirus by MOI of 50 for 24 h and maintained for another 24 h (*n*=3). **C.** Quantitative real-time PCR analysis of HDAC6 mRNA expression in SPNs transfected with HDAC6 or control shRNA lentivirus by MOI of 50 for 24h and maintained for another 24 h (*n*=3). Data, mean ± s.e.m. One way ANOVA. *, *P*<0.05.

**Supplementary Table S1**

Agents list

| **Item** | **Concentration** | **Source** |
| --- | --- | --- |
| LDN193189 | 100nM | Stemgent, MA, USA |
| SB431542 | 10μM | Amateksci, NY, USA |
| Purmorphamine | 0.65μM | Calbiochem, San Diego, CA, USA |
| MK-801 | 50, 100μM | APExBIO, Houston, TX, USA |
| CHIR-99021 | 50, 100μM | APExBIO, Houston, TX, USA |
| GSK-3β siRNA | 20, 50, 100nM | Santa Crus, CA，USA |
| HDAC6 shRNA | 1, 10, 50, 100 (MOI) | Santa Crus, CA，USA |
| Control shRNA | 1, 10, 50, 100 (MOI) | Santa Crus, CA，USA |
| Cal-520 | 1μM | Abcam |

**Supplementary Table S2**

**Antibody list**

| **Antibody** | **Isotype** | **Dilution** | **Source** |
| --- | --- | --- | --- |
| ßIII-tubulin | Mouse IgG | 1:10,000 | Sigma |
| ßIII-tubulin | Rabbit IgG | 1:10,000 | Covance Research Products |
| GABA | Rabbit IgG | 1:10,000 | Sigma |
| GABA | Mouse IgG | 1:2,000 | Sigma |
| MAP2 | Mouse IgG | 1:10,000 | Sigma |
| MAP2 | Rabbit IgG | 1:5,000 | Sigma |
| Bassoon | Mouse IgG | 1:1,000 | Abcam |
| Synaptophysin | Mouse IgG | 1:5,000 | Chemicon |
| GSK-3β | Rabbit IgG | 1:500 | Cell Signaling Technology |
| GSK-3β | Mouse IgG | 1:500 | Cell Signaling Technology |
| pGSK-3β (ser-9) | Mouse IgG | 1:500 | Cell Signaling Technology |
| HDAC6 | Rabbit IgG | 1:500 | Cell Signaling Technology |
| pHDAC6 (ser-22) | Rabbit IgG | 1:500 | Cell Signaling Technology |
| α-Tubulin | Mouse IgG | 1:200 | Cell Signaling Technology |
| β-actin | Mouse IgG | 1:1,000 | Abmart |
| GAPDH | Mouse IgG | 1:1,000 | Abmart |

| Experimental models: Cell lines | | |
| --- | --- | --- |
| H9 and H9-GFP | Dr. Zhang Suchun lab |  |
| hiPSC | Dr. Ma lab of Fudan |  |
| Hela | ATCC |  |
|  |  |  |
|  |  |  |
