# Supplementary figures and images for: Ketamine impairs growth cone and synaptogenesis in human GABAergic projection neurons via GSK-3β and HDAC6 signaling
Source: Mol Psychiatry. 2022 Nov 21;29(6):1647–59. doi: 10.1038/s41380-022-01864-5 (PMC11371642; doi:10.1038/s41380-022-01864-5)

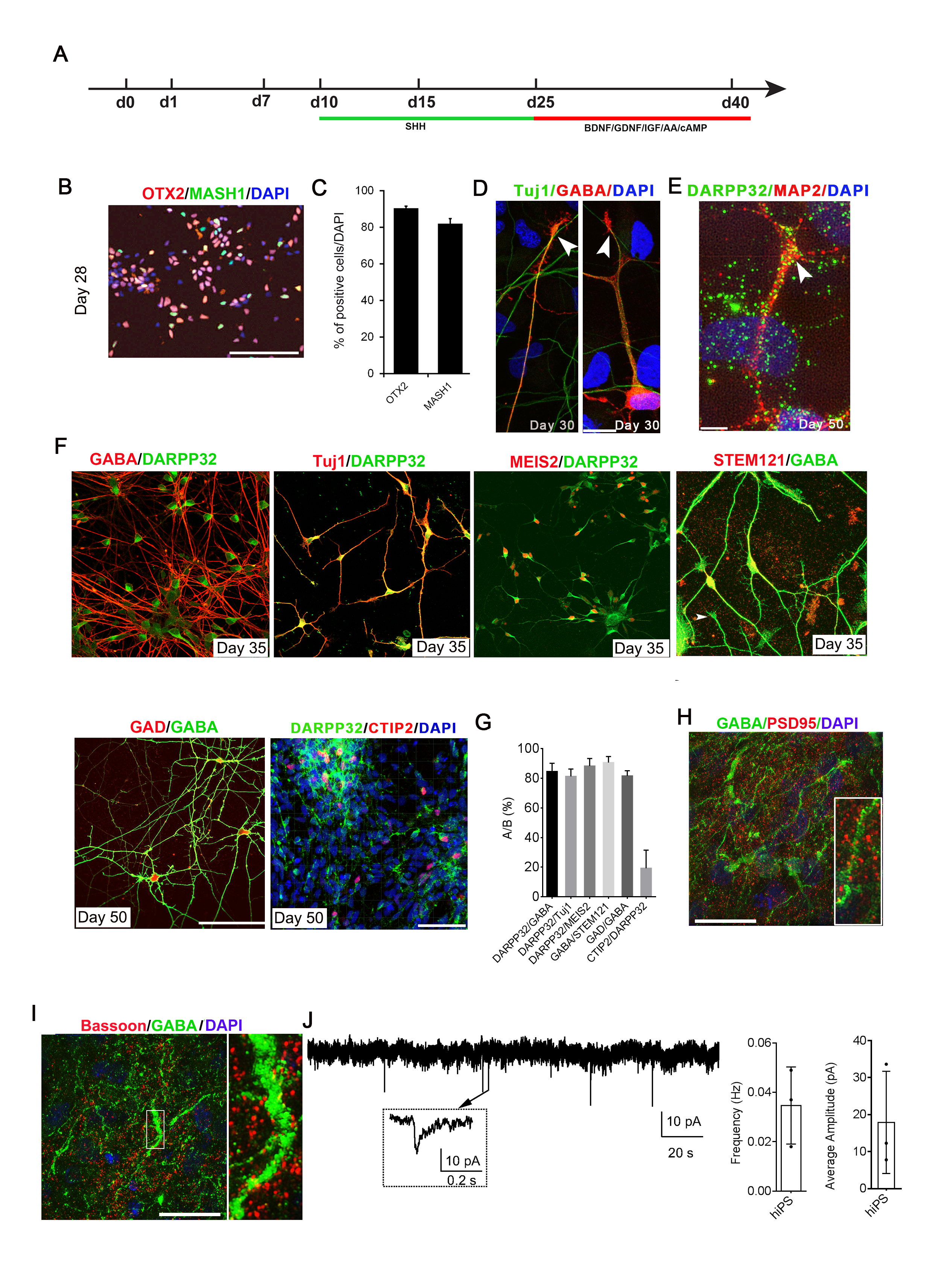

Supplement: Supplementary file 3 — Supplementary Figure 1 [file 41380_2022_1864_MOESM3_ESM.tif]

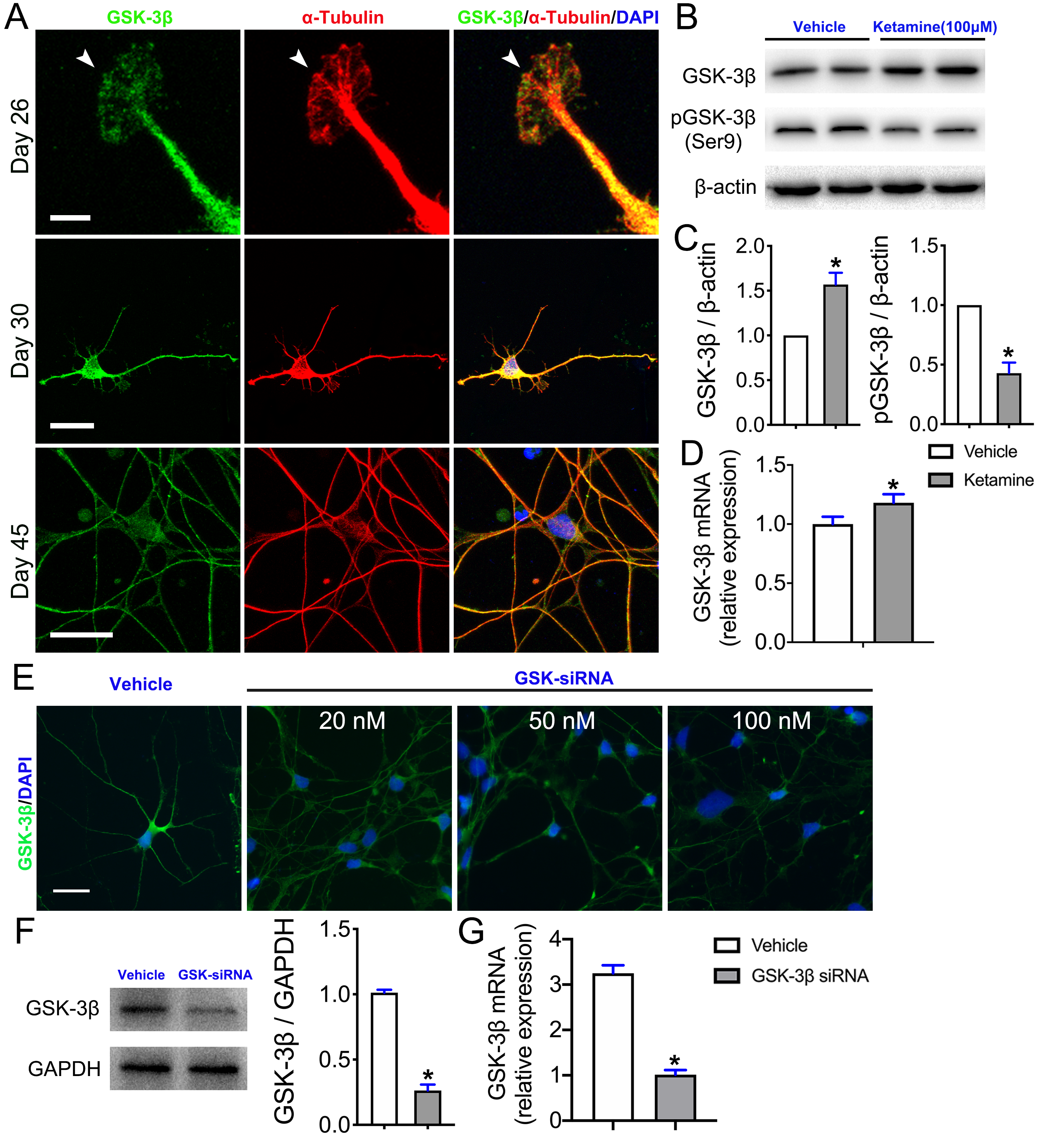

Supplement: Supplementary file 4 — Supplementary Figure 2 [file 41380_2022_1864_MOESM4_ESM.tif]

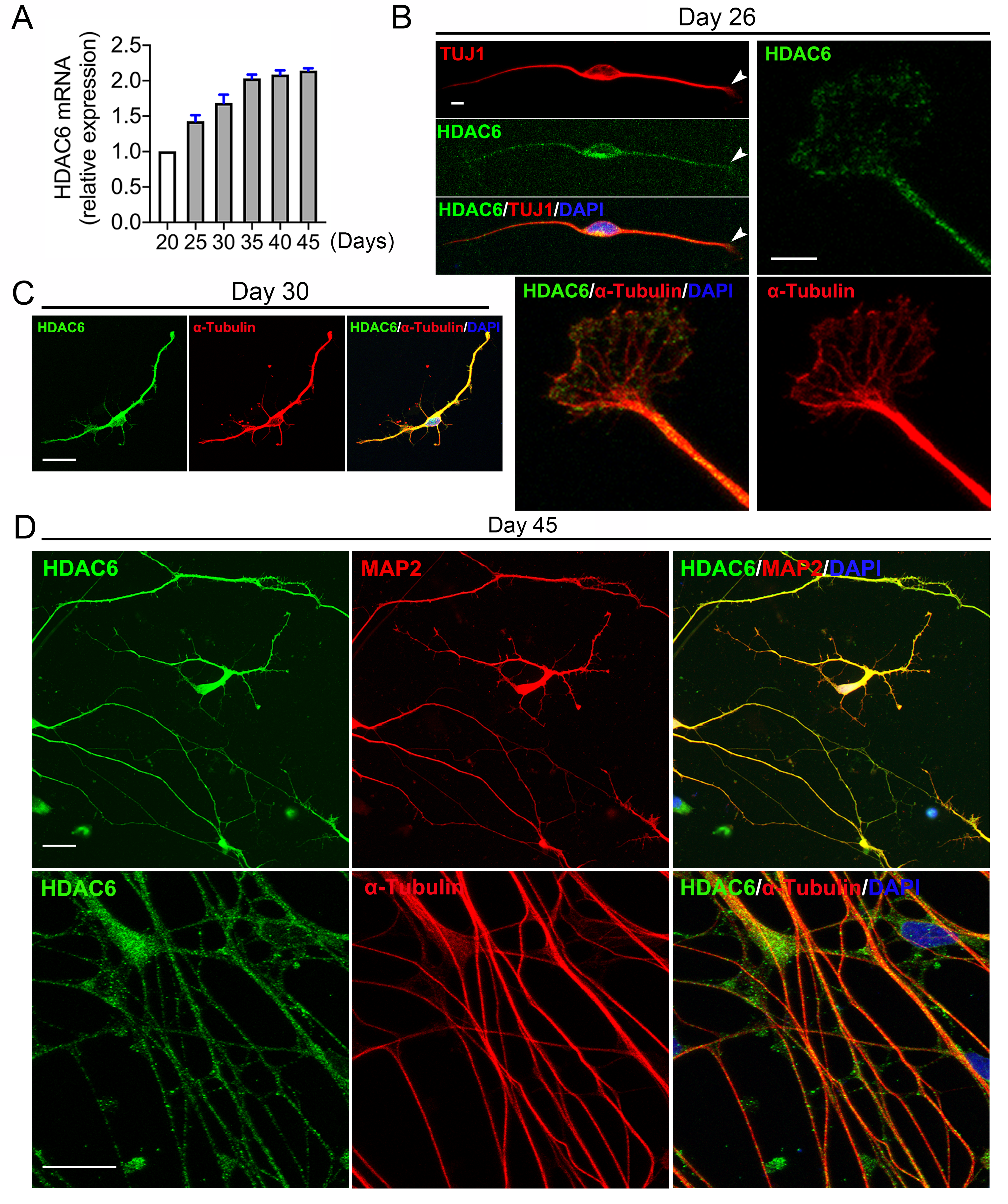

Supplement: Supplementary file 5 — Supplementary Figure 3 [file 41380_2022_1864_MOESM5_ESM.tif]

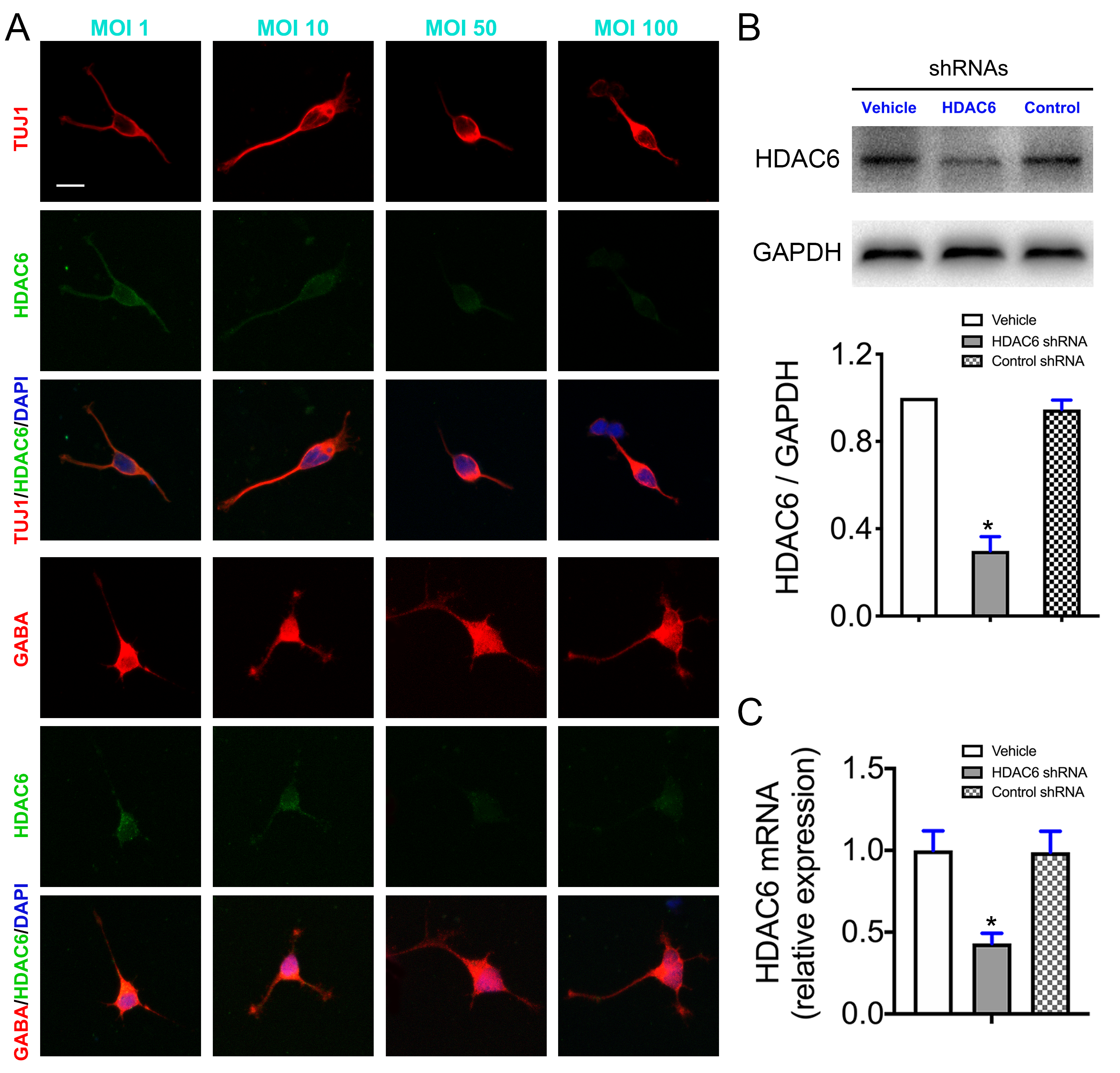

Supplement: Supplementary file 6 — Supplementary Figure 4 [file 41380_2022_1864_MOESM6_ESM.tif]
